# A case for using grid architecture for state public health informatics: the Utah perspective

**DOI:** 10.1186/1472-6947-9-32

**Published:** 2009-06-22

**Authors:** Catherine J Staes, Wu Xu, Samuel D LeFevre, Ronald C Price, Scott P Narus, Adi Gundlapalli, Robert Rolfs, Barry Nangle, Matthew Samore, Julio C Facelli

**Affiliations:** 1 Department of Biomedical Informatics, School of Medicine, the University of Utah, Salt Lake City, USA; 2 Department of Internal Medicine, School of Medicine, The University of Utah, Salt Lake City, (AG, MS), USA; 3 Utah Department of Health, Salt Lake City, USA; 4 Center for High Performance Computing, The University of Utah, Salt Lake City, USA

## Abstract

This paper presents the rationale for designing and implementing the next-generation of public health information systems using grid computing concepts and tools. Our attempt is to evaluate all grid types including data grids for sharing information and computational grids for accessing computational resources on demand. Public health is a broad domain that requires coordinated uses of disparate and heterogeneous information systems. System interoperability in public health is limited. The next-generation public health information systems must overcome barriers to integration and interoperability, leverage advances in information technology, address emerging requirements, and meet the needs of all stakeholders. Grid-based architecture provides one potential technical solution that deserves serious consideration. Within this context, we describe three discrete public health information system problems and the process by which the Utah Department of Health (UDOH) and the Department of Biomedical Informatics at the University of Utah in the United States has approached the exploration for eventual deployment of a Utah Public Health Informatics Grid. These three problems are: i) integration of internal and external data sources with analytic tools and computational resources; ii) provide external stakeholders with access to public health data and services; and, iii) access, integrate, and analyze internal data for the timely monitoring of population health status and health services. After one year of experience, we have successfully implemented federated queries across disparate administrative domains, and have identified challenges and potential solutions concerning the selection of candidate analytic grid services, data sharing concerns, security models, and strategies for reducing expertise required at a public health agency to implement a public health grid.

## Background

This paper presents the rationale for designing and implementing the next-generation state public health information systems using grid computing concepts, methods and tools. Our attempt is to evaluate all grid types including data grids for sharing information and computational grids for accessing computational resources on demand. Public health is a broad domain that requires intensive and collaborative uses of disparate and heterogeneous information to perform three primary functions: assessment, policy development, and assurance. [1] In the United States of America (US), it is the state's responsibility to see that functions and services necessary to address the mission of public health are in place. [2] Therefore, to meet the goals of health monitoring, protection, prevention, and promotion, state public health authorities are involved in diverse program areas that each have their own data sources and needs (i.e., vital records and statistics; communicable disease prevention and control; environmental health and safety; injury control; emergency and disaster preparedness; bioterrorism detection and preparedness; maternal and child health, mental health and substance abuse, chronic disease and conditions, community health assessment and surveillance, and monitoring of access, quality and cost of health care).[2] Similar functions are performed by local, regional, tribal, and national public health agencies, as well as by essential public health partners such as food, agriculture and environmental quality agencies, which create demands to integrate or share information vertically and laterally within and among public health organizations. To date, system integration and interoperability in public health has been limited. The next-generation public health information systems must overcome barriers to integration and interoperability, leverage advances in information technology, address emerging priorities, and meet the needs of all the stakeholders. Grid-based architecture provides one potential technical solution that deserves serious consideration.

Currently, state public health information systems in the United States are not well integrated. Most public health information systems consist of data silos that meet the needs of a single program and many result from past categorical funding and support from national entities, such as the US Centers for Disease Control and Prevention (CDC).[3,4] These systems often require redundant data entry and are unable to track service utilization across programs or analyze disparate data from multiple sources to quickly recognize new patterns and trends. It is widely recognized that data integration and/or system interoperability from multiple sources is critical for evidence-based public health practice and timely detection of outbreaks and threats.[5] However, integrating and sharing such data using traditional architectural models has posed a significant challenge. There are limited examples of integrated systems, including child health information systems [6] and notifiable condition reporting systems that are currently in development to improve interoperability between states and the CDC (e.g., the US National Electronic Disease Surveillance System (NEDSS).[7]

Both centralized and distributed models of data integration have been used by state public health agencies. The traditional view is that centralized models achieve superior integration. US state health departments in Missouri and Rhode Island have successfully built centralized systems. In both locations, centralized child health information systems were developed that integrated or linked birth records, immunizations, lead testing and other relevant records in a central repository. These projects successfully developed a system that benefits all participating stakeholders,[6] but are not easily extensible to meet the ever changing requirements. Most other states have made little progress in this direction. Given that state public health agencies are administrated and financed by a complex mix of federal and state categorical funding, practice under discrete legal authority and regulations, and that political leadership changes with each election cycle, most state health departments operate under a distributed organizational structure. It is difficult to develop a sustainable centralized informatics infrastructure within such a distributed organization.

At the US Utah Department of Health (UDOH), 79 silo applications and databases were identified during an inventory performed in 2002. The systems range from the Utah Statewide Immunization Information System (USIIS), a HIMSS Davies Public Health award-winning system,[8] to spreadsheets with a few hundred records for persons with infectious diseases. For a few systems, a moderate level of interoperability has been achieved. For instance, the Utah Child Health Advanced Record Management system (CHARM) developed a federated model and a service-oriented architecture to achieve interoperability among vital records, the immunization registry, the newborn hearing screening program, the baby watch/early intervention program, and the office of recovery services in the Utah Department of Human Services. In a separate communication linkage, the statewide immunization registry and the Women, Infant, and Children (WIC) system share data electronically using an internally developed application. Unfortunately, these examples of interoperability are the exceptions rather than the norm and for the most part do not provide extensible architectural models that can be easily generalized to other applications. Certainly, the integrated analyses that are necessary for evidence-based public health practice cannot be adequately supported by Utah's existing public health information system.

Efforts to construct centrally aggregated public health databases may not be the optimal solution to enable public health epidemiology and program goals. Public health data systems are widely distributed and growing in number, and there are fiscal, cultural, social and political impediments to data sharing. In addition, central aggregation is defined differently among stakeholders at the local, state and national levels. Next-generation public health information infrastructures require shared and dynamic access to tools, knowledge, standards, data, and resources to aggregate disparate, heterogeneous information to meet ever-changing user needs.

To date, grid computing technologies have successfully enabled translational clinical research and massive computational analysis of large biomedical datasets.[9] For example, the caGrid connects data and tools from over 50 disparate cancer centers and underlies caBIG, one of the most comprehensive, multi-institutional cancer research infrastructures https://cabig.nci.nih.gov/.[10] The Biomedical Informatics Research Network (BIRN, http://www.nbirn.net/) provides the infrastructure to support collaborations, data sharing and analysis tools among computer scientists, neuroscientists and engineers in the United States and the United Kingdom. GeneGrid provides a service-oriented architecture for a virtual bioinformatics laboratory focused on antibody and drug research and development.[11] The Shared Pathology Informatics Network (SPIN) provides an Internet-based virtual database for researchers to locate appropriate human tissue specimens for cancer research.[12] Using grid technologies the VOTES (Virtual Organizations for Trials and Epidemiological Studies) grid has developed a portal that provides distributed access to several databases modeling the schema and data structure in use by health organizations, such as the National Health Service in Scotland. [13]The @neurIST (Integrated Biomedical Informatics for the Management of Cerebral Aneurysms) project is an initiative to provide a grid based IT infrastructure for the management, integration and processing of data associated with the diagnosis and treatment of cerebral aneurysm and subarachnoid hemorrhage.[14]

Within the European HealthGrid initiative there are numerous projects that attempt to supports drug discovery and telediagnosis, and aims to enable radiologists from geographically dispersed hospitals to share standardized mammograms for diagnostic and epidemiologic inquiries.[15]The Globus MEDICUS project supports the federation of DICOM medical imaging devices into a healthcare grid to address image sharing, processing and archiving among providers and researchers.[16] In addition, shared resources through grid computing have allowed for parallel execution of existing algorithms and applications in such areas as mining genomic data,[17] determination of a protein secondary structure,[18] and analysis of microbial genome sequences to identify potential drug targets for new antibiotics.[19] While an extensive list of all grid systems in use today in biomedical research is outside the scope of this paper, our modest listing illustrates the diversity of biomedical informatics specialties that use grid technology to provide the scientific community with shared access to tools and resources.

Use of grid computing to meet public health needs has been increasingly explored as far as the maturity of grid technology allows it. Researchers have proposed grid computing services for geographic information systems and resource-intensive statistical analysis, [20-22] and a prototype for an epidemic surveillance system has been developed outside the US (e.g., IntegraEPI).[23] At the US national level, the CDC National Center for Public Health Informatics (NCPHI) has recently introduced the concept of a "Public Health Grid" to interconnect public health departments, regional health information systems, providers, and the National Health Information Network.[24,25]

Grid architecture is appealing for public health information system development as it promotes an open collaborative network that leverages open source software and infrastructures, enables continuing existence of legacy applications; supports a strong security model, uses standards and a service-oriented architecture, allows distributed and federated database and web services access, and enables push and pull multi-directional data exchange. The economic, social, and technological models associated with grid computing match the public health environment. However, the literature on grid architectures still lacks examples of operational or even prototype or planning efforts for public health use that would meet the needs of a state public health department.

The objectives of this paper are to describe the rationale for using grid architecture for public health, to describe candidate use cases, and to report on the initial process underway in Utah to develop a series of prototypes that could be used as proof of concept to develop a novel public health information network based on grid architecture.

## Discussion

**Figure 1:**
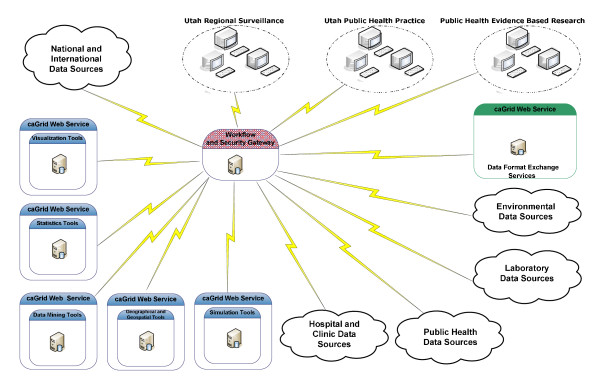
**Overall proposed architecture for the Utah public health grid demonstration projects**.

Several features of grid architecture http://www.ogf.org/ make its use attractive as a viable platform for modern public health informatics. We considered the following features to guide our selection of use cases that deserve consideration for prototype development and deployment on a Utah public health grid (Figure 1).

### A. Granularity for security and privacy

This feature allows a great deal of access control and federation of identities, which is important because public health information systems are used by numerous constituencies with varying access rights to the data. Moreover, grid architecture allows for the separation of identity management and authorization components of the system, simplifying the negotiation of data access rights by different organizations that may want to share only limited data. This architectural separation also permits the concurrent independent development of different components of the security model. Finally, the rich grid security infrastructure (GSI) allows for the rapid formation and dissolution of virtual organizations, which may be used in the formation of multidisciplinary research teams and rapid response ad hoc specialty teams.

### B. Dynamic data access

This feature eliminates the need for a monolithic data integration infrastructure and allows the rapid deployment of new applications. By treating applications and data equally, complex applications can be built by combining existing software using grid-enabled workflows.

### C. Modular and concurrent development

Since the grid architectural definition clearly specifies the interface between different components, applications and data source gateways can be developed in parallel, eliminating serious bottlenecks observed in more structured data integration architectures.

### D. Access to advanced computational resources

Traditionally, health departments have not had access to advanced computational facilities, but certainly they could benefit from using advanced analytical and modeling techniques. Many of these advanced resources are now available as grid services http://www.teragrid.org/. It will be possible to access these resources with minimal effort by grid-enabling public health applications.

### E. Integration with other Grid services

Public health analysts and first responders can greatly benefit from incorporating non-health data with traditional healthcare data to improve the detection and mitigation of events of public health significance. Weather forecasts, environmental data, earthquake damage predictions, for example, are being incorporated into emerging grids for analyses and decision-making.[26,27] Grid-enabling public health information systems will facilitate the assimilation of non-health related data, along with the analytical and modeling tools to improve public health practices and incident response.

Rather than developing a comprehensive grid-based information system to support public health activities in Utah, our approach is incremental and follows the concept of an "ecosystem" of grid components, as proposed by the Globus Alliance http://www.globus.org/grid_software/ecology.php. The use cases described here can evolve into production systems that can be incorporated into an evolving Utah public health grid, which in turn can be incorporated into the emerging national public health grid. We believe that agreement about an overall architecture that is acceptable to and fulfills the needs of all stakeholders is of utmost importance.

The overall architecture for the proposed Utah public health grid is depicted in Figure 1. In this architecture, we are treating analytical services and data sources equally. The workflow and security gateway is central to the architecture and controls access to any resources in the grid and implements access policies determined by appropriate policy makers. By using the high granularity of the grid security infrastructure (GSI), it is possible to grant access rights according to the multiple roles of the users of the system. The gateway also will implement a grid-enabled workflow system (e.g., Pegasus http://pegasus.isi.edu/; Kepler http://kepler-project.org/; or Taverna http://taverna.sourceforge.net/ to describe complex analytical or surveillance tasks that can be automatically performed, if desired. Finally, implementation of a data format exchange server is a key feature of the architecture. This engine will be able to reformat as needed all the data streams in the system. The architecture does not limit the type of data conversion tools available; the tools may implement a combination of traditional vocabulary methods or more advanced probabilistic methods or natural language processing tools. The system is highly modular so each component can be independently developed.

Grid technology is a promising technology for developing the new generation of integrated and interoperable public health surveillance and service programs at a state health agency. Grid technology principles fit the structure and management philosophy of public health in general, and UDOH in particular. In fact, the UDOH has already developed systems that follow grid principles, and we can leverage their existing "grid-friendly" practices.

However, grid technologies are not mature enough to allow for off-the-shelf implementation. Before introducing these new systems into an operational environment, public health practitioners from the UDOH and researchers from the Department of Biomedical Informatics at the University of Utah have been collaborating to develop proof of concept prototypes to demonstrate how grid technology can enhance public health surveillance and services. Three scenarios have been selected because they represent common needs and have attributes that will benefit from Grid architecture, including 1) the use of disparate and heterogeneous data, 2) the need to integrate data across jurisdictions for local and national use, 3) the presence of systems that are not scalable with their current architecture, and 4) the ongoing need for data and analytic tools. The scenarios and our approach for their grid implementation are described in the next subsections.

#### A. Integration of internal and external data sources with analytic tools and computational resources

**Figure 2:**
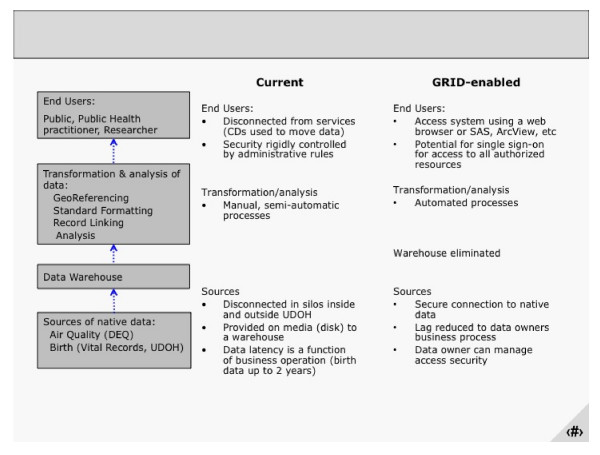
**Current and envisioned grid-enabled architecture of the environmental public health tracking network**.

Utah is one of 19 states in the United States funded to develop an Environmental Public Health Tracking Network (EPHTN) to monitor and measure health indicators related to environmental exposures, including asthma, birth defects, childhood lead poisoning, and cancer. [28] To meet this goal, the Utah EPHTN has data sharing agreements with nine state agencies. The data stores are disparate and heterogeneous and range from small Microsoft Access datasets to data stored in mainframe legacy systems both within and outside the health department information system enterprise. The current architecture illustrates the approach to centralize geo-referenced, standardized, de-identified, and aggregated data into a warehouse after applying GIS, vocabulary and security models to the data retrieved from the partners (Figure 2). The warehoused data will then be reported to a national warehouse and viewed locally using an existing query tool called IBIS-PH. The comprehensive analysis and modeling of these data using complex clustering and modeling techniques will most likely exceed the computational capacity available at the UDOH. In addition, access to timely data is limited by current business processes for transferring sets of data to UDOH.

Working in partnership with the Utah Cyberinfrastructure Council, we could make high performance computing (HPC) resources available as web services deployed using the Web Services Resource Framework (WSRF, http://www.globus.org/wsrf/) to the EPHTN program at the UDOH. Using these capabilities, the public health program at the UDOH will have access to very large computer capacity without the need to either build the capacity in-house and/or transfer the data to a HPC center outside of their control. Moreover, the use of grid-aware workflows will allow the source data providers and the UDOH to routinely perform complex modeling and analysis of these data.

#### B. Provide external stakeholders with access to public health data and services

Records of deaths, communicable diseases, and other selected events are stored and managed by state public health authorities because they have the responsibility to: 1) store historical data to maintain legal records and monitor trends, 2) verify that events meet defined criteria that can be tracked over time and across jurisdictions, and 3) implement and evaluate appropriate control measures. State-based records concerning deaths and communicable diseases are valuable to epidemiologists within Utah, but are also valuable to epidemiologists in neighboring states and national agencies, such as CDC and Homeland Security. For example, in 122 cities across the US, registrars voluntarily report directly to the CDC the total number of deaths by age group and the number of deaths for pneumonia or influenza during the previous week. This information is summarized and reported weekly in the Morbidity and Mortality Weekly Report (MMWR, http://www.cdc.gov/mmwr/preview/mmwrhtml/mm5804md.htm). The information is used by epidemiologists and others to monitor trends in total deaths and deaths due to pneumonia or influenza. According to epidemiologists in Utah, there is a need to improve data visualization, recognize aberrant patterns, and monitor trends over time for other causes of death, as needed. Similarly, a query to a neighboring state about an emerging communicable disease problem, whether it results in death or not, requires phone calls and database queries, or waiting for the information to be forwarded and aggregated at the national level. It would be beneficial to enable real-time queries of the heterogenous data, across jurisdictions, with appropriate permissions.

While a local integrated system could be developed that would serve the needs of Utah, what would happen if there were suspicion of an outbreak across jurisdictional boundaries, or a large-scale global pandemic? Grid technologies can be used to prototype public health surveillance systems that can be dynamically implemented when their need arises. To achieve this goal, it is necessary to start developing small-scale prototypes that demonstrate feasibility and identify issues to be resolved before engaging in large-scale implementations. To demonstrate grid architecture, we will encapsulate death records using the OGSA Data Architecture and implement workflow to access grid-enabled text processing and analytic tools. We will create an interface and provide access to these data at appropriate levels of detail to authorized epidemiologists.

#### C. Access, integrate, and analyze internal data for the timely monitoring of population health status and health services

The Utah Department of Health (UDOH) has developed an online Indicator-Based Information System for Public Health (IBIS-PH) to monitor information about the health status of Utahns and Utah's health care system.[29] The system has four components. The first component includes 142 online indicators available to the public and researchers. Each indicator includes a summary table or graphic based on the user's request, as well as textual information describing the definition, data sources, and other relevant information about the indicator. The second component pulls de-identified data from 17 silo databases within the UDOH enterprise for internet users to query and analyze the data. The third component is a secured query system for two public health programs to internally analyze their identifiable data. The fourth component is the secure internal administrative module. Indicator stewards can directly and manually update the data for their own indicators at any time. Both the administrators for IBIS-PH and the data suppliers must manually transfer, standardize, process, and update data from the 19 silo databases every 12 months.

To demonstrate grid architecture, we could encapsulate selected databases making them available as web services using the OGSA Data Architecture and implement a grid based workflow service application that can be used to automatically update existing indicators as well as to dynamically develop new indicators. The prototype application could be made available to interested parties in UDOH for testing, evaluation and feedback.

Since January 2008, we have gained knowledge and experience with issues that need to be addressed to implement a Utah Public Health Grid.

**Figure 3:**
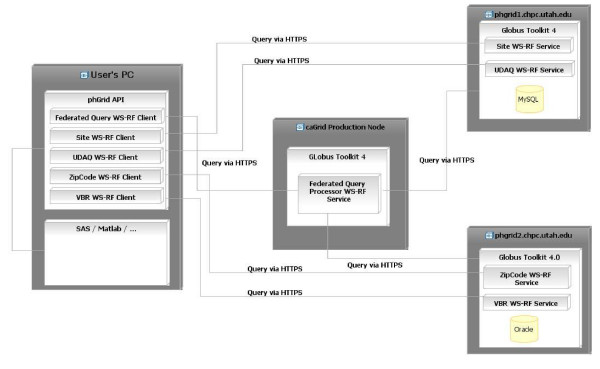
**Deployment diagram of the prototype environmental public health tracking network**.

To further our understanding of GRID in a public health environment, we created a prototype for the environmental public health tracking network (EPHTN) (Figures 2 and 3). During phase one, we successfully met our goal of creating functional federated queries that span disparate systems, as depicted in Figure 3. We created data grid services for the Vital Birth Records and ZipCode data grid services using Oracle (deployed on the grid node"phgrid1"), and Utah Department of Air Quality and Monitor Station Site Description data grid services using MySQL (deployed on the grid node "phgrid 2") and we successfully executed federated queries from a web portal (the caGRID production node). To demonstrate this capability we used a client (User's computer) that delivers the federated query to an analytical tool, in our case SAS and MATLAB.). To simulate a real word scenario the grid nodes phgrid1 and phgrid2 were deployed under two different and independent administrative domainsthat would correspond, for example, to a real case in which phgrid1 could be administered by the Utah Department of Health and phgrid2 could be administered by the US CDC. This capability was demonstrated allowing collaborators from the CDC Grid team to query data with and without the Grid security enabled. To deploy these services we used existing and evolving caGRID tools and technology that reduce the effort to implement the GRID infrastructure.

During phase two, we explored the selection of analytic grid services and attempted to implement public health-based logic that used the federated queries to allow access to the information for external users. For this purpose real de-identified public health data was needed to implement the public health logic for the planned web interface. The primary data source for air quality data is not the Utah Department of Health, the planned analysis was not routinely performed, and the security models had not yet been demonstrated to outside stakeholders. These issues led to problems with data sharing agreements and concern that information would be accessible and misinterpreted by outside users of the data.

We learned several lessons from our experience during 2008. First, the following criteria should be considered when selecting an analytic grid service: 1) identify analytic processes that may benefit from a single source code repository to minimize code maintenance efforts, 2) identify processes with a high need for internal and external reuse (e.g., geocoding and geo-referencing), and 3) avoid processes that are specific to one situation or application because they can be handled by statistical, geospatial, or other analytic tools that access data on the public health grid using an API. A candidate analytic grid service should have high potential for reuse.

Second, we identified several data sharing issues and two potential solutions. There is a need for off-the-shelf templates for data sharing contracts that can meet the needs of most situations. It may be possible to use the caBIGData Sharing and Security Framework https://cabig.nci.nih.gov/glossary/ploneglossarydefinition.2008-05-28.3668557123 and Data Sharing & Intellectual Capital https://cabig.nci.nih.gov/working_groups/DSIC_SLWG/ as a potential model. Data sharing concerns may be averted if the analytic processes can be brought to the data. Preliminary work is underway for a solution that would transport the logic composed in the service to the administrative domain of the health entity so sensitive data never leaves the health entity domain.

Third, concerns about the security of data on the grid are prevalent. Although fundamentally it is not new, the flexible and robust Grid Security Infrastructure (GSI) appears to some users to be a new security mechanism. This created vast confusion regarding how secure the grid is for public health. GSI meets the federal requirements for working with personally identified records and the NCPHI team at CDC is currently drafting a US PHGrid security policy to share with stakeholders.

Fourth, the technical expertise required to install and configure a GRID node or implement a GRID application for grid services is high and may be beyond the level of expertise typically available at a state public health department. Our technical team had over four years of experience with GRID technologies at the start of phase one. This expertise was helpful because, as early adopters, we experienced problems with inadequate documentation, evolving tools, and the need to learn a few new tools (e.g., caGrid) due to software evolution. Problems with limited expertise may be addressed by a recent effort at NCPHI to provide a phGRid installer to stand-up GRID nodes and operate certain public health applications. This tool may significantly reduce the burden and costs on public health organizations.

## Summary

Based on years of practical experience in managing information to protect the health of Utahns, the Utah public health informatics community has concluded that grid-based architectures are a promising avenue to develop a 21^st ^century public health informatics infrastructure to provide timely evidence-based public health services and policies. While we consider our conceptual approach to be correct, we are well aware of the limitations of existing implementations of grid tools. We concede that we cannot deploy a reliable public health informatics system based on grid technologies today; however, by using the highly modular characteristics of grid architectures and the principle of the "ecosystem" of grid components, we will be able to make significant progress in the near future. Our approach leverages the experience and success of other synergistic activities in Utah and elsewhere. We intend to work in parallel in three areas: securing funds, prototyping, and moving prototype systems into production. The high granularity of the grid security infrastructure (GSI) will allow us to decouple difficult data access policy issues from technical implementation issues, thereby eliminating one of the serious bottlenecks in previous attempts for data integration in comprehensive public health systems.

## Competing interests

The authors declare that they have no competing interests.

## Authors' contributions

All authors participated in the developing of the concepts described in this paper and read and approved the final manuscript.

## Pre-publication history

The pre-publication history for this paper can be accessed here:

http://www.biomedcentral.com/1472-6947/9/32/prepub
